# The association of functional polymorphisms in genes encoding growth factors for endothelial cells and smooth muscle cells with the severity of coronary artery disease

**DOI:** 10.1186/s12872-016-0402-4

**Published:** 2016-11-11

**Authors:** Tadeusz Osadnik, Joanna Katarzyna Strzelczyk, Andrzej Lekston, Rafał Reguła, Kamil Bujak, Martyna Fronczek, Marcin Gawlita, Małgorzata Gonera, Jarosław Wasilewski, Bożena Szyguła-Jurkiewicz, Marek Gierlotka, Mariusz Gąsior

**Affiliations:** 1 2nd Department of Cardiology and Angiology, Silesian Center for Heart Diseases, Marii Curie-Skłodowskiej Street 9, 41-800 Zabrze, Poland; 2 Genomics Laboratory, Kardio-Med Silesia Science and Technology Park, Marii Curie-Skłodowskiej Street 10C, 41-800 Zabrze, Poland; 3 Department of Medical and Molecular Biology, School of Medicine with the Division of Dentistry, Medical University of Silesia, Jordana Street 19, 41-808 Zabrze, Poland; 4 3rd Department of Cardiology, School of Medicine with the Division of Dentistry in Zabrze, Medical University of Silesia, Katowice, Marii Curie-Skłodowskiej Street 9, 41-800 Zabrze, Poland; 5 Silesian Center for Heart Diseases, Marii Curie-Skłodowskiej Street 9, 41-800 Zabrze, Poland

**Keywords:** Growth factors, Atherosclerosis, Coronary artery disease, Polymorphism, VEGF-A, bFGF, PDGFB, TGF-β1, EGF

## Abstract

**Background:**

Despite the important roles of vascular smooth muscle cells and endothelial cells in atherosclerotic lesion formation, data regarding the associations of functional polymorphisms in the genes encoding growth factors with the severity of coronary artery disease (CAD) are lacking. The aim of the present study is to analyze the relationships between functional polymorphisms in genes encoding basic fibroblast growth factor (bFGF, FGF2), epidermal growth factor (EGF), insulin-like growth factor-1 (IGF-1), platelet derived growth factor-B (PDGFB), transforming growth factor-β1 (TGF-β1) and vascular endothelial growth factor A (VEGF-A) and the severity of coronary atherosclerosis in patients with stable CAD undergoing their first coronary angiography.

**Methods:**

In total, 319 patients with stable CAD who underwent their first coronary angiography at the Silesian Centre for Heart Diseases in Zabrze, Poland were included in the analysis. CAD burden was assessed using the Gensini score. The TaqMan method was used for genotyping of selected functional polymorphisms in the *FGF2*, *PDGFB*, *TGFB1*, *IGF1* and *VEGFA* genes, while rs4444903 in the *EGF* gene was genotyped using the polymerase chain reaction-restriction fragment length polymorphism (PCR-RFLP) method. The associations between the selected polymorphisms and the Gensini were calculated both for the whole cohort and for a subgroup of patients without previous myocardial infarction (MI).

**Results:**

There were no differences in the distribution of the Gensini score between the genotypes of the analyzed polymorphisms in *FGF2*, *EGF*, *IGF1*, *PDFGB*, and *TGFB1* in the whole cohort and in the subgroup of patients without previous MI. The Gensini score for *VEGFA* rs699947 single-nucleotide polymorphism (SNP) in patients without previous myocardial infarction, after correction for multiple testing, was highest in patients with the A/A genotype, lower in heterozygotes and lowest in patients with the C/C genotype, (*p* value for trend = 0.013, false discovery rate (FDR) = 0.02). After adjustment for clinical variables, and correction for multiple comparisons the association between the *VEGFA* genotype and Gensini score remained only nominally significant (*p* = 0.04, FDR = 0.19) under the dominant genetic model in patients without previous MI.

**Conclusions:**

We were unable to find strong association between analyzed polymorphisms in growth factors and the severity of coronary artery disease, although there was a trend toward association between rs699947 and the severity of CAD in patients without previous MI.

**Electronic supplementary material:**

The online version of this article (doi:10.1186/s12872-016-0402-4) contains supplementary material, which is available to authorized users.

## Background

Early atherosclerotic changes in coronary arteries are characterized by proteoglycans, extracellular lipid accumulation and a large number of vascular smooth muscle cells (VSMCs) that migrate from the medial portion of the artery into the endothelial layer [1]. At later stages, changes may include an increase in collagen production, which takes place within a lesion with a large number of VSMCs, and a lesion may evolve into a thick cap fibroatheroma [2]. However, depletion of smooth muscle cells (SMCs) may also occur, along with a decrease in collagen production and an increase in lipid accumulation, leading to the creation of vulnerable plaque or a thin cap fibroatheroma [3]. Thin cap fibroatheromas are more susceptible to erosion/ruptures that lead to acute coronary syndromes, whereas thick cap fibroatheromas are mostly responsible for stable coronary artery disease (CAD) symptoms [2, 3]. Despite the important role of VSMCs in atherosclerotic lesion formation, thus far, there have been no studies, with the exception of analyses regarding vascular endothelial growth factor A (VEGF-A) [4] and transforming growth factor-β1 (TGF-β1) [5], analyzing functional polymorphisms in genes encoding growth factors that affect VSMCs in terms of the severity of coronary atherosclerosis. The aim of the present study is to analyze the relationships between functional polymorphisms in genes encoding basic fibroblast growth factor (bFGF, FGF2), epidermal growth factor (EGF), insulin-like growth factor-1 (IGF-1), platelet derived growth factor-B (PDGFB), TGF-β1 and VEGF-A and the severity of CAD in patients with stable CAD undergoing their first coronary angiography.

## Methods

In total, 319 patients with stable CAD who underwent their first coronary angiography at the Silesian Centre for Heart Diseases in Zabrze, Poland, were enrolled. All patients were subjected to implantation of at least one bare metal stent. CAD burden was assessed using the Gensini score, which is a derivative of the dominance of the right or left coronary artery and of the degree of stenosis [6]. The analysis of lesion severity was performed with quantitative coronary angiography (QCA) and the narrowing between 0–25 %, 25–50 %, 50–75 %, 75–90 %, 90–99 %, and 100 %; stenosis was scored as, 1, 2, 4, 8, 16, and 32 points, respectively [6]. Cardiologists assessing coronary angiography records were blinded to genotypic results, and researchers performing genotypic analysis were blinded to angiographic results. Analyses were performed in the whole cohort and in the subgroup of patients without previous myocardial infarction (MI).

### Genotyping

Genomic DNA from 200 μl of whole blood was extracted using the commercially available GeneMatrix Quick Blood DNA Purification Kit (EURX, Poland) according to the manufacturer’s instructions. The eluted DNA was precipitated and dissolved. The aim was to discriminate between two alleles of specific single-nucleotide polymorphisms (SNPs) of *FGF2* (rs308395), *EGF* (rs4444903), *IGF1* (rs35767), *PDGFB* (rs2285094), *TGFB1* (rs1800470) and *VEGFA* (rs699947).

### Genotyping of the EGF gene using PCR-RFLP

The *EGF* gene was genotyped using the polymerase chain reaction-restriction fragment length polymorphism (PCR-RFLP) using the primers (nucleotide position −78 to +164) and methods with minor modification described by Shahbazi et al. [7]. PCR reaction mixtures with a final volume of 25 μl contained the following: 2 μl of genomic DNA, 0.2 μl of 25 mM dNTP Mix (containing dATP, dCTP, dGTP, and dTTP), 1.2 μl of 10 μM forward and reverse primers (Genomed, Poland), 2.5 μl of DyNAzyme Buffer (Thermo Scientific, USA), 0.3 μl of DyNAzyme II DNA Polymerase (2 U/μl; Thermo Scientific, USA) and 17.6 μl of molecular grade water (Qiagen, Germany). The PCR reaction was performed in a Mastercycler personal thermocycler (Eppendorf, Germany), and the conditions were 5 min of initial denaturation at 95 °C, followed by 35 cycles of denaturation at 95 °C for 45 s, annealing at 57 °C for 45 s, extension at 72 °C for 45 s and a final extension at 72 °C for 10 min. The length of the amplified EGF fragment was 242 bp. The PCR product was digested using AluI to type the EGF 61A/G polymorphism by RFLP analysis. The PCR product was digested at 37 °C for 12 h in a Mastercycler personal thermocycler (Eppendorf, Germany) in a solution containing 5 μl of PCR product, 1 μl of AluI restriction enzyme (10 U/μl, Thermo Scientific, USA), 1 μl of 10× Buffer Tango (ThermoScientific, USA), and 3 μl of molecular grade water (Qiagen, Germany) per reaction. Then, AluI was inactivated by incubation at 65 °C for 20 min. AluI cleaved the 242-bp PCR product into fragments of 15, 34 and 193 bp for GG homozygotes and 15, 34, 91 and 102 bp for AA homozygotes.

### Genotyping the FGF2, PDGFB, TGFB1 and VEGFAA genes using the TaqMan assay

DNA samples were genotyped by employing the 5′ nuclease assay for allelic discrimination using TaqMan SNP Genotyping Assays (Applied Biosystems, CA, USA). The PCR reaction mixtures included 5 ng of genomic DNA in a 25-μl reaction volume and the following concentrations of other reagents: 2× TaqMan Genotyping Master Mix (No AmpErase UNG), primers at 900 nM final concentration and 200 nM of each probe (Applied Biosystems). Samples which have three different genotypes were amplified and subjected to sequencing and, after confirming the genotype, used as positive control samples. Sequence analysis of a polymorphic region of the genes were performed by using BigDye® Terminator v3.1 Cycle Sequencing Kit (Applied Biosystems, Thermo Fisher Scientific, USA). The reaction was carried out in a capillary sequencer 3730xl DNA Analyzer. As negative control DNase-, RNase-, and Protease - free Water (Qiagen, Germany) was used. We were able to establish genotype of rs1800470 (TGF-β1) for 316 (99.1 %) patients, whilst genotypes of rs308395 (bFGF2), rs4444903 (EGF), rs35767 (IGF-1), PDGFB (rs2285094) and VEGF-A (rs699947) were successfully established for all patients.

Reactions were performed in 96-well plates. Control samples were run in parallel with unknown DNA samples. The samples were read on a 7300 Real-Time PCR System and were analyzed using SDS 1.4 software (Applied Biosystems). Additionally, 10 % of the samples were repeated for quality control with complete congruence. Details are presented in Table 1.

**Table 1:** Sequences of oligonucleotide probes used for polymorphism detection

Gene	Polymorphism	Nucleotide base	Context sequence
*FGF2*	rs308395	C/G	CTCTTCTATGGCCTACTTTCTACTG[C/G] TATTTGTGTTACTCATGCTACCCAT
*IGF1*	rs35767	A/G	GAATTTTTTCTTTTTTTTTTTTTCC[A/G] CATGACTCTCAGGGGACTGACACAT
*PDGFB*	rs2285094	T/C	CAACCAATAGAGGGGCCAATAGAAC[T/C] GCCCAGCTGAGCCCAGTCAACCCCC
*TGFB1*	rs1800470	A/G	TAGCCACAGCAGCGGTAGCAGCAGC[A/G] GCAGCAGCCGCAGCCCGGAGGGCGG
*VEGFA*	rs699947	A/C	GCCAGCTGTAGGCCAGACCCTGGCA[A/C] GATCTGGGTGGATAATCAGACTGAC

The research was carried out according to The Code of Ethics of the World Medical Association (Declaration of Helsinki). Informed consent was obtained from all the examined patients. The study was approved by the Ethics Committee of Silesian Medical Chamber, Katowice, Poland.

### Statistical analysis

Continuous variables are presented in terms of medians and interquartile ranges, as they were not normally distributed, and dichotomic variables are presented as percentages. Genotype frequencies were determined by their direct summing. Fisher’s exact test was used for determination of consistency with Hardy–Weinberg equilibrium (HWE). The Kruskal-Wallis test was used to test for differences in the Gensini score according to the genotypes of the studied polymorphisms. The Jonckheere-Terpstra test was used to analyze possible trends in Gensini score distribution. The associations between the Gensini score and the polymorphisms were analyzed in the whole cohort and in the subgroup of patients without previous MI, using the following genetic models: dominant, recessive and log-additive for *EGF*, *IGF1*, *PDGFB*, *TGFB1* and *VEGFA*, and dominant only for *FGF2* due to the low number of minor homozygotes. These models were adjusted for clinical variables that reached *p* values < 0.3 in the univariate regression analysis with the Gensini score as an outcome variable. This cut off point was chosen as *p* values higher than 0.3 as selection criteria for adjusting for potential confounders are very unlikely to improve model selection [8]. The associations of the observed polymorphisms with the Gensini score after adjustment were analyzed, and false discovery rate (FDR) was calculated to account for multiple comparisons. The number of patients enrolled is sufficient to detect, with a power of 80 % the differences of Gensini score of 14.8 point or greater assuming group sample sizes of 60 and 250 with a standard deviation for both groups of 29.00 and at FDR = 0.05. Analyses were completed using NCSS (Number Crunching Statistical Systems, Kaysville, Utah, USA) [9] software, PASS (Power Analysis and Sample Size Software. NCSS, LLC. Kaysville, Utah, USA) [10] and with the SNPassoc [11] and fdrtool package in the R environment [12, 13].

## Results

The baseline clinical and demographic features of the study population and﻿ the subgroup of patients without previous MI are ﻿shown in Table 2. Patients’ genotypes were distributed in accordance with Hardy-Weinberg equilibrium, with the exception of the rs4444903 polymorphism (Table 3). Minor allele frequencies (MAFs) were similar to those reported in other European populations, with the exception of rs308395 in the *FGF2* gene (Table 3). The associations between *FGF2*, *EGF*, *IGF1*, *PDGFB*, *TGFB1*, and *VEGFA* genotypes and the severity of CAD were investigated in the whole cohort and in the subgroup of patients without previous MI (Table 4). In general, there were no differences in the distribution of the Gensini score between the genotypes of the analyzed polymorphisms *in FGF2*, *EGF*, *IGF1*, *PDFGB*, and *TGFB1* in the whole cohort and in the subgroup of patients without previous MI. Similarly, after adjustment for clinical variables, there was no significant association between the Gensini score and polymorphisms in *FGF2*, *EGF*, *IGF1*, *PDFGB*, and *TGFB1* under dominant, recessive, and log-additive models (Additional file 1: Table S1 and Additional file 2: Table S2).

**Table 2:** Clinical characteristics of the study population and the subgroup without MI

Variable	Whole cohort *n* = 318	Patients without previous MI *n* = 249
Age (years)	64 (57 ÷ 70)	64 (58 ÷ 71)
Male sex, *n* (%)	224 (70.4)	171 (68.7)
Hypertension, *n* (%)	225 (70.8)	185 (74.3)
Heart failure, *n* (%)	72 (22.6)	53 (21.3)
Ejection fraction, *n* = 284 (89 %)	50 (45.0 ÷ 55.0) *	52 (48 ÷ 55) **
Atrial fibrillation, *n* (%)	46 (14.5)	40 (16.1)
Hypercholesterolemia, *n* (%)	150 (47.2)	117 (47.0)
Previous MI, *n* (%)	69 (21.7)	–
Diabetes, *n* (%)	75 (23.6)	59 (23.7)
Diet/oral pharmacotherapy	45 (13.8)	38 (15.2)
Insulin	31 (9.7)	21 (8.4)
Creatinine (μmol/l)	79.8 (67.0 ÷ 89.9)	79.8 (66.5 ÷ 91.2)

*n = 284 (89.3 %) ** *n* = 221 (88.8 %); *MI* myocardial infarction

**Table 3:** MAFs of the analyzed SNPs in the analyzed cohort and the European Union (EU) population

Gene	Polymorphism	Alleles	MAF	MAF EU Population [52]	HWE *P* value
*FGF2*	rs308395	C/G	9.6 %	16 %	0.51
*EGF*	rs4444903	A/G	42.0 %	39 %	0.006
*IGF1*	rs35767	A/G	16.0 %	16 %	0.06
*PDGFB*	rs2285094	T/C	40.3 %	46 %	0.48
*TGFB1*	rs1800470	A/G	43.7 %	38 %	0.57
*VEGFA*	rs699947	A/C	49.8 %	50 %	0.07

**Table 4:** Associations between genotype and the Gensini score in the whole cohort and in patients without previous MI

		Genotype			*P* value Kruskal-Wallis	*P* value Trend test
		C/C	C/G	G/G		
*FGF2*	Whole cohort	25 [16–48]	24 [14–59]	33.5 [22.8–45.5]	0.91	0.35*
	Without previous MI	22 [15–44]	23.5 [14–56.8]	27 [20–34]	0.80	0.28*
		A/A	A/G	G/G		
*EGF*	Whole cohort	24 [15–56.5]	26 [15.5–49.5]	22.3 [16–43.3]	0.79	0.34**
	Without previous MI	22.5 [14–44]	25 [14–46]	20.5 [15.3–42.5]	0.68	0.34**
		A/A	A/G	G/G		
*IGF1*	Whole cohort	26 [20–44]	24.5 [14–44.3]	25 [16–56]	0.53	0.18*
	Without previous MI	26.5 [21.9–44]	23 [12–43]	22 [14.5–46]	0.35	0.31*
		C/C	T/C	T/T		
*PDGFB*	Whole cohort	22.8 [16–46]	25.5 [14–52]	25.5 [16–52.3]	0.78	0.25*
	Without previous MI	22.3 [16.0–45.5]	22.5 [13–44.8]	22.0 [16.0–44]	0.75	0.32*
		A/A	A/G	G/G		
*TGFB1*	Whole cohort	24 [14–46]	25 [16–47]	26 [16–58]	0.5	0.14*
	Without previous MI	24 [13.8–44.5]	22 [15.3–39.8]	22.5 [15.8–49.3]	0.96	0.45*
		A/A	A/C	C/C		
*VEGFA*	Whole cohort	27 [17.5–46.3]	26 [16.0–56]	22 [14–47]	0.2	0.048**
	Without previous MI	25 [17–44.5]	24.5 [14–46.4]	18 [12.0–43.3]	0.055	0.013**

Jonckhere Terpstra test for trend: *increasing, **decreasing

For the *VEGFA* rs699947 SNP in the total patient group, the median Gensini score was highest in patients with the A/A genotype, lower in heterozygotes and lowest in patients with the C/C genotype, with nominal *p* value for trend = 0.04 and FDR = 0.07. This trend was significant for adjusted threshold of significance after excluding patients with previous MI (nominal *p* value = 0.013, FDR = 0.02). After adjustment for clinical variables, the association between the *VEGFA* genotype and the Gensini score did not meet the threshold of significance under the dominant genetic model in patients without previous MI (nominal *p* value = 0.04, FDR = 0.19) (Table 5).

**Table 5:** Association of the *VEGFA* genotype with the Gensini score after adjustment for clinical variables

Gene /polymorphism	Dominant Model* (mean Gensini score ± standard error)		*P*	Recessive Model* (mean Gensini score ± standard error)		*P*	Log additive model*	*P*
	Genotypes			Genotype			Difference in Gensini score per minor allele (95 % CI)	
	A/A (ref.)	A/C + C/C		A/A + A/C (ref.)	C/C		Per C allele	
	Whole cohort							
*VEGFA* rs699947	37.9 ± 3.2	35.6 ± 1.9	0.57	37.9 ± 2.0	31.6 ± 2.7	0.22	−2.3 (−6.4 ÷ 1.9)	0.28
	Patients without previous myocardial infarction							
*VEGFA* rs699947	26.4 ± 2.4	35.1 ± 2.1	0.04	31.4 ± 2.0	35.5 ± 3.3	0.41	3.8 (−0.51 ÷ 8.0)	0.09

*The models were adjusted for: age, sex, hypertension, atrial fibrillation, diabetes mellitus, previous myocardial infarction and creatinine

## Discussion

The pivotal roles of growth factors for endothelial cells (ECs) and for SMCs have been recognized for over 20 years [14]. Growth factors such as bFGF, EGF, IGF-1, PDGFB, TGF-β1 and VEGF-A play important roles in VSMC and EC migration and proliferation, therefore playing important roles in the pathogenesis of atherosclerosis. Polymorphisms selected for this study was based on their functionality, which was understood as influencing the level of gene expression or given growth factor concentration (rs699947, rs4444903, rs180047) or on the association of a given polymorphism with other conditions (rs308395, rs699947, rs2285094) [15]. The growth factors that we analyzed in this paper are both acting on and secreted by ECs and VSMCs [16].

The role of bFGF in atherosclerosis is associated with its mitogenic activity and its role in angiogenesis [17]. Additionally, bFGF leads to a decrease in collagen [18–20] and an increase in metalloproteinase (MMP) synthesis [21], which may be one of the elements playing roles in vulnerable plaque formation [22]. The localization of rs308395 within the promoter region of the *FGF2* gene might explain its association with the *FGF2* expression level [23, 24]. In a Polish population, the C/C genotype of rs308395 predicted a more aggressive course of non-Hodgkin lymphoma [23]. In our paper, we did not find any association between the severity of CAD and the rs308395 polymorphism.

rs444903 in the *EGF* gene is the second of the analyzed polymorphisms. EGF influences the atherosclerotic process because it affects the structure of atherosclerotic lesions. Similarly to bFGF, EGF hampers the synthesis of collagen fibers (type I and III) and increases *MMP*-*9* and *MMP*-*1* gene expression levels [25]. Studies on primates have shown that a high-cholesterol diet leads to activation of signal pathways associated with EGF [26]. Similarly, as is the case for rs308395 in the *FGF2* gene, rs444903 is associated with the course of many cancers, including lung cancer [27–29]. Despite the role of EGF in atherosclerosis, our paper is the first to assess the association of the known functional polymorphism rs444903 with the severity of coronary atherosclerosis. Nonetheless, we did not find any association between analyzed polymorphism of *EGF* and Gensini score in patients with CAD who underwent their first coronary angiography.

IGF-1 is also one of the EC and SMC growth regulators. IGF-1 level has been inversely linked with atherosclerosis [30], and rs35767 polymorphisms in the *IGF1* gene are associated with IGF-1 serum level [31]. Previously, we studied the relationship between *IGF1* gene polymorphisms and in-stent restenosis in patients undergoing bare metal stent implantation, but we did not find any association [32]. In contrast to polymorphisms in *FGF2* and *EGF*, polymorphism rs35767 in the *IGF1* gene was studied in the context of atherosclerosis; however, data are scarce [31]. Sesti G. et al. showed that rs35767 was associated with not only IGF-1 level but also intima-media thickness [31]. Another paper analyzed the association between polymorphisms in the *IGF1* gene and coronary arteriosclerosis. Similar to our results, the authors did not observe any significant correlation between rs35767 and the severity of CAD (measured with the Gensini score) [33].

PDGF is a growth factor for cells of mesenchymal origin, including VSMCs, and supports their migration to the lumens of vessels. *PDFGB* expression was also elevated in patients with diabetes. With the exception of one study in an African population, there have been no analyses of *PDGFB* polymorphisms in the context of atherosclerosis. There was, however, one study associating rs2285094, which is located near the splice site, with higher incidence of sclerodermia [34]. This polymorphism was also tested for possible associations with type 1 diabetes [35] and IgA nephropathy [36], though no relationship was found. We also did not detect any association between rs2285094 and CAD burden.

The role of TGF-β1 in atherosclerosis has been confirmed and is well studied. In general, TGF-β1 stimulates extracellular matrix production and inhibits SMC and EC proliferation [5, 37, 38]. However, at levels exceeding 1–2 fg/cell, TGF-β1 may stimulate SMC, fibroblast and chondrocyte proliferation [39]. TGF-β1 is also found in blood serum, although studies aiming to compare TGF-β1 levels and CAD severity yielded conflicting results [5, 40–42]; additionally, TGF-β1 levels depend on genetic factors [43–46]. Some researchers have associated the C alleles with higher TGF-β1 levels [44–46], whereas other authors have postulated higher levels in T/T homozygotes [44]. In our study, we did not observe any relationship between rs1800470 in the gene coding for TGF-β1 and the severity of coronary artery disease.

VEGF-A is a growth factor for EC and a factor influencing migration of SMCs. The migration of SMCs over the basal membrane to the intima is stimulated by growth factors that influence the change of the SMC phenotype from contractile to proliferative [14]. SMCs that migrate to the intima proliferate and secrete extracellular matrix proteins that form a fibrous cap on the atherosclerotic lesion. Enlargement of atherosclerotic lesions leads to local hypoxia, which leads to increased VEGF-A synthesis and neovascularization of the atherosclerotic lesion. Studies on animal models have shown that VEGF-A injection caused increases in the atherosclerotic lesion area and influenced the lipid profile and lipoprotein lipase concentration [47]. VEGF-A levels are also genetically determined; rs699947 is one of the polymorphisms that significantly influences its serum concentration [48, 49]. This polymorphism was linked to increased risk of various neoplasms [50], including lung cancer [51]. In our paper, we have shown trend toward association between rs699947 and the severity of CAD in patients without previous MI.

Similar results were obtained by Howell et al., who showed a significant correlation between rs699947 and the severity of CAD, but only among patients with stable CAD and without previous MI [4]. Howell et al. explained this finding by the fact that the influence of rs699947 on atherosclerosis is modest and can be veiled by stronger environmental or genetic factors leading to MI. While agreeing with the interpretation of Howell et al., we are also of the opinion that different results in patients with previous MI and in patients with stable CAD without previous MI might stem from different pathophysiologies leading to the development of stable and vulnerable plaques [16].

## Conclusions

We were unable to find strong association between analyzed polymorphisms in growth factors and the severity of coronary artery disease, although there was a trend toward association between rs699947 and the severity of CAD in patients without previous MI.

## Additional files

Additional file 1: Table S1. Associations of genotype with the Gensini score in the whole cohort. (DOCX 14 kb)
Additional file 2: Table S2. Associations of genotype with the Gensini score among patients without previous MI. (DOCX 14 kb)

## Abbreviations

bFGF: Basic fibroblast growth factor
CAD: Coronary artery disease
ECs: Endothelial cells
EGF: Epidermal growth factor
IGF-1: Insulin-like growth factor-1
MAF: Minor allele frequency
MI: Myocardial infarction
MMPs: Matrix metalloproteinases
PDGF: Platelet derived growth factor
TGF-β1: Transforming growth factor-β1
VEGF-A: Vascular endothelial growth factor A
VSMCs: Vascular smooth muscle cells
