# Supplementary material for: The association of functional polymorphisms in genes encoding growth factors for endothelial cells and smooth muscle cells with the severity of coronary artery disease
Source: BMC Cardiovasc Disord. 2016 Nov 11;16:218. doi: 10.1186/s12872-016-0402-4 (PMC5106826; doi:10.1186/s12872-016-0402-4)
Supplement: Additional file 1: Table S1. — Associations of genotype with the Gensini score in the whole cohort. (DOCX 14 kb) [file 12872_2016_402_MOESM1_ESM.docx]

**Table S1. Associations of genotype with the Gensini score in the whole cohort.**

| Gene  /Polymorphism | Dominant Model*  (mean Gensini score ± standard error) | | P | Recessive Model*  (mean Gensini score ± standard error) | | P | Log additive Model* | P |
| --- | --- | --- | --- | --- | --- | --- | --- | --- |
|  | Genotypes | |  | Genotype | |  | Difference in Gensini score per minor allele (95%CI) |  |
|  | C/C (ref.) | C/G + G/G |  | C/C + C/G (ref.) | G/G |  | Per G allele |  |
| *FGF2*  rs308395 | 35.6 ± 1.8 | 38.8 ± 5.6 | 0.17 | ------------------ | -------------- | ---- | ------------------------- | ---- |
|  | A/A (ref.) | A/G + G/G |  | A/A + A/G (ref.) | G/G |  | Per G allele |  |
| *EGF*  rs4444903 | 36.5 ± 2.6 | 36.1 ± 2.1 | 0.91 | 37.1 ± 1.9 | 32.9 ± 3.2 | 0.22 | -1.6 (-5.7 ÷ 2.5) | 0.46 |
|  | G/G (ref.) | A/G + A/A |  | G/G + A/G (ref.) | A/A |  | Per A allele |  |
| *IGF1*  rs35767 | 38.2 ± 2.1 | 31.0 ± 2.2 | 0.23 | 36.5 ± 30.3 | 30.3 ± 3.7 | 0.49 | -3.6 (-9.2 ÷ 2.1) | 0.22 |
|  | T/T (ref.) | T/C + C/C |  | T/T + T/C (ref.) | C/C |  | Per C allele |  |
| *PDGFB*  rs2285094 | 38.2 ± 3.0 | 35.1 ± 1.9 | 0.32 | 36.8 ± 1.8 | 32.8 ± 3.5 | 0.38 | -2.7 (-7.2 ÷ 1.82) | 0.24 |
|  | A/A (ref.) | A/G + G/G |  | A/A +A/G (ref.) | G/G |  | Per G allele |  |
| *TGFB1*  rs1800470 | 35.2 ± 3.0 | 36.9 ± 2.0 | 0.91 | 35.5 ± 1.8 | 40.4 ± 4.1 | 0.25 | 1.3 (-3.2 ÷ 5.8) | 0.57 |

*The models were adjusted for: age, sex, hypertension, atrial fibrillation, diabetes mellitus, previous myocardial infarction and creatinine.
