# Supplementary material for: The association of functional polymorphisms in genes encoding growth factors for endothelial cells and smooth muscle cells with the severity of coronary artery disease
Source: BMC Cardiovasc Disord. 2016 Nov 11;16:218. doi: 10.1186/s12872-016-0402-4 (PMC5106826; doi:10.1186/s12872-016-0402-4)
Supplement: Additional file 2: Table S2. — Associations of genotype with the Gensini score among patients without previous MI. (DOCX 14 kb) [file 12872_2016_402_MOESM2_ESM.docx]

**Table S2. Associations of genotype with the Gensini score among patients without previous MI.**

| Gene  /Polymorphism | Dominant Model*  (mean Gensini score ± standard error) | | P | Recessive Model*  (mean Gensini score ± standard error) | | P | Log additive Model* | P |
| --- | --- | --- | --- | --- | --- | --- | --- | --- |
|  | Genotypes | |  | Genotype | |  | Difference in Gensini score per minor allele (95%CI) |  |
|  | C/C (ref.) | C/G + G/G |  | C/C + C/G (ref.) | G/G |  | Per G allele |  |
| *FGF2*  rs308395 | 31.8 ± 1.8 | 36 ± 4;3 | 0.14 | ------------- | ----------- | ------ | ------------------- | ---- |
|  | A/A (ref.) | A/G + G/G |  | A/A + A/G (ref.) | G/G |  | Per G allele |  |
| *EGF*  rs4444903 | 32.5 ± 2.7 | 32.7 ± 2.2 | 0.96 | 33.6 ± 2.0 | 28.9 ± 3.1 | 0.24 | -1.5 (-5.9 ÷ 2.8) | 0.49 |
|  | G/G (ref.) | A/G + A/A |  | G/G + A/G (ref.) | A/A |  | Per A allele |  |
| *IGF1*  rs35767 | 34.3 ± 2.2 | 28.5 ± 2.2 | 0.23 | 32.6 ± 1.8 | 31.6 ± 3.7 | 0.66 | -3.3 (-9.0 ÷ 2.4) | 0.26 |
|  | T/T (ref.) | T/C + C/C |  | T/T + T/C (ref.) | C/C |  | Per C allele |  |
| *PDGFB*  rs2285094 | 34.5 ± 3.2 | 31.6 ± 2.0 | 0.44 | 32.9 ± 1.9 | 30.9 ± 3.7 | 0.64 | -2.0 (-6.8 ÷ 2.9) | 0.43 |
|  | A/A (ref.) | A/G + G/G |  | A/A +A/G (ref.) | G/G |  | Per G allele |  |
| *TGFB1*  rs1800470 | 33.8 ± 3.0 | 32.2 ± 2.1 | 0.54 | 32.8 ± 1.9 | 32.2 ± 3.5 | 0.94 | -0.91 (-5.6 ÷ 3.8) | 0.71 |

*The models were adjusted for: age, sex, hypertension, atrial fibrillation, diabetes mellitus, previous myocardial infarction and creatinine.
